# Cadmium-Induced Structural Destabilization of the Insulin–Zinc Complex Underlies the Paradox of Elevated but Dysfunctional Insulin

**DOI:** 10.1007/s12011-026-05255-7

**Published:** 2026-07-29

**Authors:** Diana Moroni-González, Israel Quiroga-Montes, Fabián Galindo-Ramírez, Celso Enrique Cortes-Romero, Eduardo Brambila, Victor Enrique Sarmiento-Ortega, Samuel Treviño

**Affiliations:** 1 Laboratory of Metabolomic and Chronic Degenerative Diseases, Physiology Institute, Meritorious Autonomous University of Puebla, Prol. de la 14 Sur 6301, Ciudad Universitaria, Puebla, C.P. 72560 Mexico; 2 Popular Autonomous University of the State of Puebla (UPAEP) A.C, 21 Sur #1103, Barrio de Santiago, Puebla, C.P. 72410 México; 3 Physiology Institute, Meritorious Autonomous University of Puebla, Prol. de la 14 Sur 6301, Ciudad Universitaria, Puebla, C.P. 72560 Mexico; 4 Faculty of Nursing, Meritorious Autonomous University of Puebla, Av. 25 Pte. 1304, C.P. 72410 Puebla, Mexico; 5 Faculty of Medicine, Meritorious Autonomous University of Puebla, Av. 25 Pte. 1304, Puebla, C.P. 72410 Mexico; 6 Laboratory of Chemical-Clinical Investigations, Department of Clinical Chemistry, Chemistry Department, Meritorious Autonomous University of Puebla, 14 Sur. FCQ1, Ciudad Universitaria, Puebla, C.P. 72560 Mexico

**Keywords:** Cadmium toxicity, Insulin-Cd complex, Hyperinsulinemia, Insulin dysfunction

## Abstract

Chronic cadmium (Cd) exposure, even in environmental conditions, has been associated with metabolic disorders such as dyslipidemia, insulin resistance, and type 2 diabetes. However, the molecular mechanisms underlying Cd-induced endocrine–metabolic disruption remain unclear. Using a multidisciplinary approach that combines in vivo, in vitro, and in silico analyses, we examined the effects of environmentally relevant Cd concentrations on lipids and glucose homeostasis, β-cell proliferation, insulin secretion, and the biofunctionality of insulin-Cd complex. Male Wistar rats were exposed orally to CdCl₂ (15 or 32 ppm) for three months. zoometric, glycemic, and lipid parameters were evaluated, along with markers of glucose-stimulated insulin secretion (GSIS), β-cell proliferation (Ki-67), and survival (Bcl-2). Crystallographic, spectroscopic (UV–Vis, FTIR), and in silico analyses characterized the coordination chemistry of Zn²⁺ and Cd²⁺ within insulin complexes, and the biological activity of insulin-Cd was assessed in diabetic models. Cd exposure produced a dose-dependent increase in zoometric and biochemical parameters, accompanied by hyperinsulinemia and impaired β-cell function. Isolated islets showed exaggerated second-phase insulin release and increased Ki-67 with reduced Bcl-2 immunoreactivity, suggesting compensatory β-cell proliferation under subtoxic stress. Spectroscopic and theoretical analyses support the possibility that Cd²⁺ substitutes Zn²⁺ within insulin coordination sites, leading to structural perturbations. In vivo, insulin–Cd conjugates exhibited diminished hypoglycemic efficacy despite elevated circulating insulin levels, indicating reduced bioactivity. In conclusion, Cd exposure at the experimental concentrations used in this study is associated with structural perturbations of insulin and markedly reduced biological activity, providing a plausible mechanism for Cd-induced metabolic dysfunction.

**Supplementary Information:**

The online version contains supplementary material available at https://doi.org/10.1007/s12011-026-05255-7.

## Introduction

Previously, we reported that antioxidative defense and heat shock proteins prevent pancreatic cell death, particularly in Langerhans islet cells, in an animal model exposed to a cadmium (Cd) dose corresponding to the lowest observed adverse effect level (LOAEL), under chronic conditions [1]. In toxicological risk assessment, the no observed adverse effect level (NOAEL) is defined as the highest tested dose at which no statistically or biologically significant adverse effect is detected, whereas the LOAEL corresponds to the lowest tested dose at which such an effect is observed; both are experimentally derived points of departure (PODs) obtained directly from toxicological studies. Minimal Risk Levels (MRLs) are regulatory health guidance values derived from these PODs by applying uncertainty factors that account for interspecies and intraspecies variability [2, 3].

The Cd concentrations used in the present study (15 and 32 ppm, or mg/L, in drinking water) correspond to NOAEL- and LOAEL-equivalent experimental doses previously characterized by our research group for chronic metabolic toxicity in Wistar rats [1, 4–8]. These concentrations were selected because they reproduce, in a controlled animal model, the internal Cd burden associated with prolonged low-level environmental exposure, and not because they are numerically equivalent to the tolerable intake values established for humans and expressed as µg/kg body weight/day; the latter are POD-derived regulatory limits intended for human risk assessment and cannot be directly extrapolated to dietary Cd concentrations administered to rodents [2, 3, 9]. Despite this difference in units and derivation, both experimental and regulatory approaches address the same toxicological concern, namely the exceptionally long biological half-life of Cd in humans, exceeding two decades, owing to its progressive accumulation in multiple tissues, including the liver, kidneys, bones, testis, adipose tissue, and pancreas [9]. Environmental Cd exposure occurs primarily through contaminated food and water and tobacco smoke, and has been associated with a wide spectrum of health effects [10, 11]. Various organizations have established the tolerable intake level of Cd at 0.36–0.83 µg/kg body weight/day [3, 12]; nevertheless, the health risk associated with chronic low-dose Cd exposure remains underappreciated, since intakes within this nominally tolerable range can still produce measurable metabolic disturbances in experimental models, supporting the translational relevance of the exposure scenario used here.

Emerging evidence links Cd exposure with metabolic diseases, including impaired glucose tolerance, dyslipidemia, insulin resistance, and an increased risk of type 2 diabetes; these associations are supported by both epidemiological observations and experimental studies [7, 13–19]. Mechanistically, Cd behaves as an endocrine disruptor of carbohydrate metabolism, interfering with insulin signaling and glucose homeostasis at multiple regulatory levels [20]. In rodent models, sub-chronic Cd exposure produces a characteristic metabolic fingerprint, including disrupted lipid and amino acid metabolism, oxidative stress, and impaired antioxidant gene expression mediated by the Nrf2 pathway, accompanied by histopathological damage to the liver, kidney, and pancreatic islets [21, 22]. Consistently, Cd-induced disruption of carbohydrate and lipid metabolic pathways has been associated with impaired glucose tolerance and insulin resistance in independent rat models, effects that can be partially reversed by antioxidant-rich treatments, further supporting oxidative stress as a central mechanism linking Cd exposure to metabolic dysfunction [23]. Collectively, these findings reinforce the notion that Cd is not merely a cytotoxic agent but an active contributor to the pathogenesis of metabolic disease.

In the pancreas, insulin-producing β-cells are a critical target of Cd toxicity. High doses of Cd, acute or chronic, have been shown to induce β-cell apoptosis, reduce insulin synthesis, and impair glucose-stimulated insulin secretion, largely via oxidative stress, mitochondrial dysfunction, and activation of pro-apoptotic pathways [14, 24–27]. Beyond the well-documented cytotoxic effects of Cd, evidence indicates that this metal can also induce cellular proliferation in different tissues, depending on the dose and duration of exposure [28–30]. However, our previous work demonstrated that chronic oral Cd exposure to low doses triggered adaptive responses, preventing β-cell death through antioxidative defenses and heat shock protein activity, and induced cellular proliferation [1, 13].

Therefore, the current results reveal a paradoxical scenario in which Cd exposure is associated with enhanced β-cell proliferation and increased insulin synthesis and release. Nonetheless, secreted insulin appears to be functionally impaired [13]. This phenomenon underscores a complex duality in which Cd simultaneously promotes islet survival and hyperinsulinemia, yet may compromise the hormone’s biological activity. This apparent contradiction suggests that insulin secreted under Cd-exposure conditions at low doses may exhibit structural alterations that reduce its bioactivity, possibly because Cd exhibits ionic mimicry, thereby displacing zinc (Zn) from the insulin hexamer and impairing the hormone’s structural stability and secretory capacity, given that Zn plays a central role in insulin storage, stabilization, and secretion [31].

Within β-cell secretory granules, insulin is stored as a hexamer coordinated by two Zn ions bound to histidine residues (HisB10) from three insulin monomers, forming a highly ordered quaternary structure that ensures hexamer stability and regulated release [32, 33]. Upon secretion, environmental changes promote the dissociation of the hexamer into active monomers that bind to receptors [33]. At the molecular level, the structural similarity between the two ions raises the possibility of Zn displacement by Cd, which then coordinates with histidine side chains. Each possible substitution could perturb the geometry and stability of the complex, alter the microenvironment of key aromatic residues, and produce conformational changes in the peptide backbone [34–36].

Despite the biological plausibility of these conformational and structural modifications, direct evidence linking Cd exposure to structural alterations in insulin remains limited. The present study aimed to elucidate the biochemical, structural, and functional consequences of chronic Cd exposure on the insulin–zinc complex and pancreatic β-cell physiology. Using a multidisciplinary approach that combines in vivo, in vitro, and in silico analyses, we examined the effects of environmentally relevant Cd concentrations on glucose metabolism, insulin secretion, and islet cell proliferation, as well as on the coordination chemistry and conformational stability of the insulin-Cd complex.

## Materials and Methods

### Animals and Treatment

Fifty male Wistar rats (initial weight range: 70–80 g) were obtained from the “Claude Bernard” Animal Facility at the Universidad Autonoma de Puebla (Puebla, Mexico). The animals were housed under controlled environmental conditions (22 °C; 12 h light/dark cycle) with free access to standard laboratory chow and drinking water. They were maintained on a balanced diet (5001, LabDiet; St. Louis, MO, USA) until attaining approximately 200 g in body weight. The animals were randomly assigned using a random number generator in Excel; the procedure was performed by the veterinary staff responsible for the rats to ensure blinding.

Rats were used in two independent, but complementary experimental protocols designed to address different aspects of the study hypothesis. Experimental protocol 1 (*n* = 30) was designed to evaluate the metabolic consequences of chronic Cd exposure, including glucose homeostasis, insulin secretion, β-cell proliferation, and structural characterization of insulin. And experimental protocol 2 (*n* = 20) consisted of an independent alloxan-induced type 1 diabetes model that was used exclusively to evaluate the in vivo biological activity of the experimentally generated insulin–Cd conjugate compared with insulin–Zn.

For experimental protocol 1, the rats were allocated into a control group (*n* = 10) and an experimental group (*n* = 20). The control group (*n* = 10) received Cd-free drinking water *ad libitum*, whereas the experimental groups received cadmium chloride (CdCl₂) at 15 ppm (*n* = 10) or 32 ppm (*n* = 10) in drinking water. Both control and experimental animals were assessed after a 3-month exposure period. At the conclusion of the exposure stage, animals underwent a 4–5-hour fasting period, and an oral glucose tolerance test (OGTT) was performed. An anhydrous glucose solution was administered orally via a gavage cannula (1.75 g/kg body weight). Then, blood samples were obtained from the tail vein at 0, 30, 60, and 90 min., under deep anesthesia, induced with a xylazine/ketamine mixture (20/137 mg/kg). Blood was drawn completely 4 h after the OGTT by cardiac puncture. Serum was separated by centrifugation and stored at − 70 °C until further analysis. The pancreas was carefully dissected, rinsed with isotonic saline to remove residual blood, and then frozen at − 70 °C or fixed in 10% neutral-buffered formalin for histopathological processing.

For experimental protocol 2, 20 male Wistar rats (280–300 g) were used; 10 animals served as controls (*n* = 10) and were injected intraperitoneally with a vehicle solution. The remaining ten rats were injected intraperitoneally with alloxan (150 mg/kg), followed by 3 days of glucose-supplemented water to stabilize hyperglycemia and hypoinsulinemia. Rats were maintained on Humalog^®^ (0.5 U/100 mg body weight) until used in the experimental protocol. The model was validated when rats presented hyperglycemia (≥ 300 mg/dL) and hypoinsulinemia (≤ 5 µU/mL). All procedures complied with the ethical requirements of the Mexican Official Standard NOM-062-ZOO-1999 and were approved by the Institutional Animal Care and Use Committee (CICUAL: VIEP/DGI/0017/2023). The study adhered strictly to both national and international regulations for the care and use of laboratory animals (ARRIVE 2.0 and NHI guidelines), with all possible measures taken to minimize the number of animals used and to reduce pain, stress, and discomfort during the experimental protocol.

### Morphometric and Biochemical Assays

Body weight, abdominal circumference, body mass index (BMI), and body fat percentage were determined. Body weight was recorded using a digital scale (Torrey, Model LPCR-20/40; Querétaro, Mexico). Size was measured from the base of the tail to the tip of the nose, using a flexible measuring tape. Abdominal circumference was obtained by measuring the distance between the diaphragm and the inguinal fold. BMI was calculated as body weight (g) divided by body length (cm²). Fat percentage was estimated according to the Lee index for rodents [6], using the formula: % fat = [(body weight (g)^0.33) / body length (mm)] × 100.

Serum levels of glucose, triglycerides (TG), total cholesterol (Chol), and cholesterol fractions [high-density lipoprotein (HDL) and low-density lipoprotein (LDL)] were quantified spectrophotometrically using commercial assay kits in an automated spectrophotometer (A-25 BioSystems, Guadalajara, Mex.). Very-low-density lipoprotein (VLDL) concentration was calculated according to Sampson’s formula [37]. Serum insulin concentration was measured with commercial ELISA kits (Merck Millipore; Toluca, Mexico) and read on a Stat Fax 2600 microplate reader at 415 nm (WinerLab Group, Buenos Aires, Argentina). Serum-free fatty acid (FFA) quantification was performed as previously described [6].

We used the mathematical homeostasis model assessment β% index (HOMA-β%) to pancreatic β-cell function in vivo, following the formula: HOMA β (%) = (360 × insulin [µU/mL])/(glucose [mg/dL] − 63) [38]. The insulinogenic index (IGI) was calculated from OGTT and insulinic response as the difference between insulin levels at 30 min and fasting, divided by the corresponding difference in glucose, that is, IGI = (Insulin 30 [µU/mL] min – Insulin 0 min [µU/mL])/(Glucose 30 min [mg/dL] – Glucose 0 min [mg/dL]). Both indices are widely validated markers of β-cell function.

### Insulin Secretion and β-Cell Responsiveness

For static glucose-stimulated insulin secretion (GSIS) in vitro, islets of Langerhans were isolated as described previously [1]. After euthanasia, immediately, the pancreas from five rats per group was aseptically distended via common bile duct cannulation (27-gauge needle) with cold HBSS containing collagenase type V (2 mg/mL; C9263, Sigma Aldrich, Mexico). Tissues were digested at 37 °C for 9 min, the reaction was stopped with cold HBSS, and the suspension was sequentially washed and centrifuged. The pellet was layered over Histopaque 1.119 and 1.077, overlaid with HBSS, and centrifuged at 900 g for 20 min to separate islets from acinar tissue. Islets were manually counted under a light microscope. Following islet isolation, groups of 50 size-matched islets were incubated overnight in RPMI medium supplemented with 11 mM glucose, 10% FBS, and 0.1% Pen/Strep to stabilize them. The next day, RPMI was replaced with sterile 2 mM glucose Krebs–Ringer buffer for 30 min before stimulation. Glucose was then added to a final concentration of 16 mM, and medium aliquots were collected at 0, 2, 15, 30, and 60 min for insulin quantification as previously described.

Immunofluorescence for islet insulin was performed on tissue sections after antigen retrieval and blocking with 2% IgG-free bovine serum albumin (BSA; Sigma-Aldrich, Mexico). Sections were incubated overnight at 4 °C with primary antibodies against insulin (1:250; ab7842, Abcam, Mexico)., After PBS washes, Rhodamine conjugated secondary antibody (Santa Cruz Biotechnology, USA) was applied for 2 h at room temperature. Nuclei were counterstained with DAPI (Vectashield, Vector Laboratories, USA), and sections were sealed. Images were acquired with a Leica fluorescence microscope and analyzed using Leica IM1000 software.

### Proliferation and Survival in Pancreatic Islets

Immunohistochemistry for Ki-67 and Bcl-2 islet, was performed on tissue sections after antigen retrieval and blocking with 2% IgG-free BSA, followed by endogenous peroxidase inhibition, samples were permeabilized with 0.2% Triton X-100, rinsed with phosphate-buffered saline (PBS), and incubated overnight at 4 °C with primary antibodies of Ki-67 (1:250; ab16667, Abcam, Mexico) or Bcl-2 (1:250; ab182858, Abcam, Mexico) After PBS washes, a peroxidase-conjugated secondary antibody (1:500; ab6908, Abcam, Mexico) was applied for 2 h at room temperature, followed by additional PBS washes. Immunoreactivity was visualized using 3,3′-diaminobenzidine, and photomicrographs at 400× magnification were captured with a brightfield microscope equipped with a camera (Leica Microsystems GmbH, Wetzlar, Germany). Semi-quantitative analysis was performed using ImageJ v1.53 (NIH, USA) to measure pixel intensity, delineate islets of Langerhans, and express the results as optical density (OD) in arbitrary units (AU). The results are shown as AU for each islet of Langerhans (100 islets).

### Structural Changes in Insulin-Cd Conjugated

For the preparation of insulin binding to Zinc or Cd, commercial Humalog^®^ insulin, which contains zinc-coordinated insulin, was used. To obtain metal-free insulin, 0.5 g of Chelex resin was hydrated in 1 mL of Tris-HCl (5 mM, pH 8.4) for 8 h under constant rotation, then centrifuged (400 g, 20 min). The hydrated beads were incubated with 1 mL of Humalog^®^ for 24 h. The mixture was centrifuged (1,200 g, 10 min), and the supernatant containing metal-free insulin was collected. For insulin–Cd construction, CdCl₂ solution (5 mg in 10 mL water) was added to the metal-free insulin (obtained from Humalog demetalization) and incubated under agitation for 48 h to achieve Cd saturation. The resulting preparations were used in subsequent assays.

To confirm metal coordination and characterize the complexes, insulin–Zn and insulin–Cd solutions were crystallized. 1 mL of metal-free insulin was mixed with HCl (0.02 M, 350 µL), CdCl₂ or ZnCl₂ (0.6 M, 35 µL), trisodium citrate (0.2 M, 175 µL), acetone (105 µL), and deionized water (34 µL), adjusting pH to 5.8 for Cd or 7.0–7.8 for Zn. Solutions were left at room temperature for 4 days and at 4 °C for 23 days until crystal formation, and analyzed by optical microscopy (400× magnification) with a brightfield microscope equipped with a camera (Leica Microsystems GmbH, Wetzlar, Germany).

For UV absorption spectroscopy of insulin preparations, Humalog^®^, Chelex-treated insulin (metal-free), Insulin–Zn, and Insulin–Cd solutions were analyzed by UV spectrophotometry at the 200–300 nm range. Absorbance spectra were recorded using quartz cuvettes and an appropriate blank. Data were collected at 1–2 nm intervals, and spectral profiles were compared to assess structural changes associated with metal removal or coordination. For FTIR spectroscopy, crystals of all insulin preparations were mixed with KBr, dried at 105 °C, ground to a fine powder, and pressed into pellets, which were analyzed on a PerkinElmer Frontier FTIR spectrophotometer (400–4000 cm⁻¹). Spectral peaks were compared with reference tables for interpretation.

### In-Silico Analysis

Three-dimensional molecular models were generated to elucidate the coordination behavior of Cd^2+^ and Zn^2+^ with insulin fragments and to assess their potential effects on the hormone’s supramolecular organization. The initial coordinates of the protein fragments were extracted from the crystal structure of the human insulin hexamer (PDB code 1EVR) [39]. The selected fragments correspond to regions containing histidine residues involved in metal coordination (HisB10). Molecular models were constructed, including two or three histidines and one metal ion (Cd²⁺ or Zn²⁺), preserving the spatial arrangement observed in the crystallographic structure, in the Gabedit software [40]. Geometry optimization and theoretical frequency calculations were performed using the MOPAC2016 package [41]. The PM7 Hamiltonian was used in all calculations, including solvent effects through the COSMO model with a dielectric constant corresponding to water. For large systems, the MOZYME localized molecular orbital method was employed to accelerate convergence. Bond order and molecular orbital analysis were used to evaluate the nature and strength of Cd–N interactions with histidine residues. Comparative analyses were performed between Cd- and Zn-coordinated models and with a control system devoid of any metal ion (Chelex-treated model). The resulting structural parameters and theoretical absorbance trends were interpreted in the context of the experimental spectra obtained for Humalog^®^, insulin–Zn, and insulin–Cd samples.

### In Vivo Evaluation of Insulin-Cd

According to experimental protocol 2 (see 2.1), an OGTT was performed on the control and type 1 diabetes (T1D) model under fasting conditions. Immediately, 8U of insulin-Cd (*n* = 5 per group) or insulin-Zn (Humalog^®^) was administered subdermally (*n* = 5 per group). Blood samples were obtained from the tail vein at 0, 30, 60, and 90 min. After OGTT, rats were anesthetized with ketamine and xylazine (20/137 mg/kg, intraperitoneally), sacrificed, and perfused with cold isotonic saline; then, the pancreas was excised, rinsed with cold saline to remove residual blood, and fixed in neutral-buffered formalin for histological processing and immunohistochemistry to evaluate insulin expression in the islets of Langerhans. Finally, glucose and insulin concentrations were quantified as described above.

### Statistical Analysis

Data are expressed as mean ± standard error of the mean (SEM). Normality of the distributions was assessed using the Shapiro–Wilk test. For comparisons among groups, one-way analysis of variance (ANOVA) was performed. To control for Type I error, post hoc analysis was performed using Bonferroni’s correction for pairwise comparisons against the control group. For data that did not follow a normal distribution (semi-quantitative data), the Kruskal–Wallis H-test was performed, followed by a post-hoc analysis with a Bonferroni correction. For multi-time-point data (glucose and insulin curves), a Two-Way Repeated Measures ANOVA was conducted to evaluate treatment effects, followed by Tukey’s post hoc test. All statistical analyses were carried out using GraphPad Prism version 8 (GraphPad Software Inc., USA). Differences were considered with a significance level of *p* ≤ 0.05. Statistical comparisons were performed against the control group.

## Results

### Morphometric and Metabolic Alterations Associated with Cadmium Exposure

Chronic exposure to Cd at doses of 15 ppm and 32 ppm significantly altered the metabolic and morphometric profiles of Wistar rats, as summarized in Table 1. Rats Cd-exposed to 15 ppm and 32 ppm showed significant dose-dependent increases in the body weight (6.6% and 13.6%; F_2,27_ = 13.8, *p* < 0.001), abdominal circumference (20.1% and 29.6%; F_2,27_ = 310.9, *p* < 0.001), BMI (27.8% and 55.6%; F_2,27_ = 84.4, *p* < 0.001), and body fat percentage (10.6% and 15.04%; F_2,27_ = 152.6, *p* < 0.001) compared to the control group, reflecting a shift toward a pro-obese phenotype induced by the metal. The highest F-scores indicate that Cd exposure has a very strong and consistent impact on zoometric markers. Likewise, we analyzed the metabolic Cd-exposure impact on glucose (45% and 73.2%; F_2,27_ = 192.3, *p* < 0.001), insulin (30.2% and 84.5%; F_2,27_ = 62.9, *p* < 0.001), triglycerides (54.3% and 76.2%; F_2,27_ = 43.8, *p* < 0.001), total cholesterol (20.5% and 24.3%; F_2,27_ = 20.2, *p* < 0.001), LDL (77.9% and 94.4%; F_2,27_ = 38.6, *p* < 0.001), and FFA (179% and 821.1%; F_2,27_ = 334.7, *p* < 0.001), which increased in both groups Cd-exposed. For most parameters, including glucose and insulin, there are significant differences not only between the control and Cd groups but also between the two Cd doses. The HDL was the only parameter to decrease by 14.6% (15-ppm) and 30.6% (32-ppm), with F_2,27_ = 16.6, *p* < 0.001. Meanwhile, although VLDL was elevated by 42.4% and 76.4% in Cd-exposed rats, it showed no significant variation across groups (F_2,27_ = 1.5, *p* = 0.253), suggesting it may be a less sensitive marker of Cd-induced metabolic disruption in this model.

**Table 1:** Morphometric and serum metabolic profile

	Control (*n* = 10)	Cd-15 ppm (*n* = 10)	Cd-32 ppm (*n* = 10)	F-score (DF_2,27_)	*p*
Weight (g)	355.6 ± 10.2	379 ± 6.8	404 ± 5.1	13.8	< 0.001*
Abdominal Circumference (cm)	17.9 ± 0.2	21.5 ± 0.1	23.2 ± 0.3	310.9	< 0.001*
BMI	0.90 ± 0.05	1.15 ± 0.02	1.4 ± 0.03	84.4	< 0.001*
Body fat %	35.9 ± 0.1	39.7 ± 0.3	41.3 ± 0.5	152.6	< 0.001*
Glucose (mg/dL)	95.2 ± 4.4	138 ± 2.5	164.9 ± 3.0	192.3	< 0.001*
Insulin (µUI/mL)	11.6 ± 0.7	15.1 ± 0.4	21.4 ± 1.5	62.9	< 0.001*
Triglycerides (mg/dL)	55.1 ± 5.6	85 ± 3.5	97.1 ± 2.1	43.8	< 0.001*
Chol (mg/dL)	80.2 ± 3.7	96.7 ± 2.6	99.7 ± 2.1	20.2	< 0.001*
HDL (mg/dL)	40.9 ± 1.5	31.9 ± 2.1	25.9 ± 1.9	16.6	< 0.001*
LDL (mg/dL)	24.9 ± 1	44.3 ± 2.9	48.4 ± 2.1	38.6	< 0.001*
VLDL (mg/dL)	14.4 ± 4.9	20.5 ± 3.8	25.4 ± 1.5	1.5	0.253
FFA (mg/dL)	1.9 ± 0.05	5.3 ± 0.9	17.5 ± 0.65	334.7	< 0.001*

The results shown are the average of 10 different experiments ± SEM. (*) indicates a significant difference with respect to control groups, as determined by a one-way ANOVA followed by a Bonferroni post-test. *BMI* Body mass index, *Chol* Total cholesterol, *HDL* cholesterol in high-density lipoprotein, *LDL* cholesterol in low-density lipoprotein, *VLDL* cholesterol in very low-density lipoprotein, *FFA* Free fatty acids

### Insulin Secretion and β-Cell Responsiveness after Cadmium Exposure

Insulin responsiveness in Cd-exposed animals was assessed using an exogenous stimulus, such as an OGTT. Under fasting conditions, the highest F-scores were observed for both parameters (glucose: 1843.85; insulin: 158.98), indicating that the Cd groups already had significantly different metabolic baselines. While all-time points remained significant, the F-score for glucose decreased at 60 min (10.88), corresponding to the point at which the control group reached its peak and began converging toward the Cd groups’ levels, suggesting a loss of insulin sensitivity. Cadmium exposure dose-dependently increased insulin levels across all time points. The Cd groups maintained the highest insulin concentrations throughout the 90 min (F_2,27_ = 158.98, 37.06, 10.16, and 155.54), suggesting severe compensatory hyperinsulinemia or impaired insulin clearance (Table 2). Two-Way Repeated Measures ANOVA and Tukey’s post-hoc analysis demonstrated a high interaction between variables (time and Cd-exposure) and significant differences for the OGTT (F_2,6_ = 82.21; *p* < 0.0001), while for insulin responsiveness, the interaction was F_2,6_ = 18.92; *p* < 0.001. In both analyses, Cd exposure was a stronger predictor.

**Table 2:** Oral glucose tolerance and insulinic responsiveness in Cd-exposed rats

	Control (*n* = 10)	Cd-15 ppm (*n* = 10)	Cd-32 ppm (*n* = 10)	F-score (DF_2,27_)	*p*
Glucose 0 min (mg/dL)	92.4 ± 1.1	141 ± 1.1	173.3 ± 1.2	1843.85	< 0.0001*
Glucose 30 min (mg/dL)	127.0 ± 1.5	150.7 ± 2.9	182.7 ± 2.5	100.69	< 0.0001*
Glucose 60 min (mg/dL)	160.7 ± 7.0	131.4 ± 2.0	162.5 ± 2.2	10.88	< 0.01*
Glucose 90 min (mg/dL)	113.0 ± 3.5	128.3 ± 1.5	134.0 ± 3.2	26.85	< 0.001*
Insulin 0 min (µUI/mL)	10.6 ± 0.4	16.1 ± 0.3	23.7 ± 0.9	158.98	< 0.0001*
Insulin 30 min (µUI/mL)	16.0 ± 0.6	18.2 ± 1.0	26.0 ± 0.6	37.06	< 0.001*
Insulin 60 min (µUI/mL)	11.8 ± 0.5	16.2 ± 0.6	20.0 ± 1.7	10.16	< 0.01*
Insulin 90 min (µUI/mL)	9.8 ± 0.2	14.5 ± 0.4	19.0 ± 1.2	155.54	< 0.0001*

The results shown are the average of 10 different experiments ± SEM. (*) indicates a significant difference with respect to control groups, F-score and p-value for each time point based on a One-Way ANOVA comparing the three groups (Control, Cd-15 ppm, and Cd-32 ppm) at each specific interval, followed by a Bonferroni’s post hoc test. Statistical inference was determined by a Two-Way Repeated Measures ANOVA followed by a Tukey’s post hoc test for interaction across time and Cd as variables

**Fig. 1:**
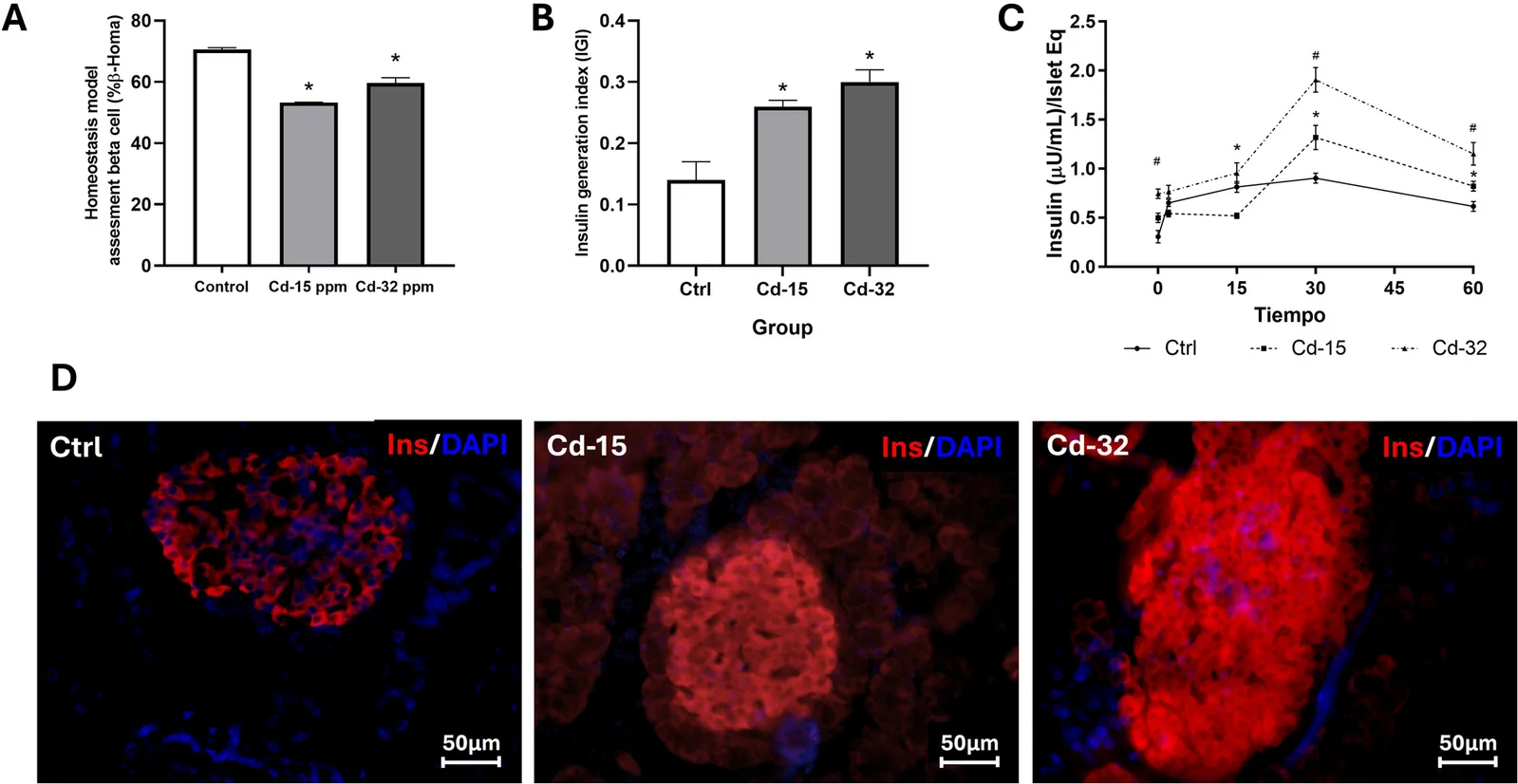
Insulin Secretion and β-Cell Responsiveness after Cadmium Exposure. (**A**) In vivo Homeostasis model assessment beta cell (HOMA-%β); (**B**) *In vivo I*nsulin generation index (IGI); (**C**) In vitro insulin secretion assays in islets with glucose (*n* = 50 islets). (**D**) Insulin immunofluorescence in Langerhans islet, insulin (red), nuclei (DAPI, blue) with a 40X objective. The data presented are the mean of the 5 repeated measurements ± SEM (standard error of the mean). For HOMA-%β and IGI, a One-Way analysis of variance (ANOVA) using Bonferroni’s post hoc was performed. For the in vitro insulin secretion assay, a Two-Way Repeated Measures ANOVA was conducted to evaluate Cd dose effect, followed by Tukey’s post hoc test. (*) Indicates a significant difference at *p* ≤ 0.05

Given the combination of hyperinsulinemia and hyperglycemia in Cd-exposed animals, HOMA-β% was quantified (Fig. 1A). The results showed that β-cells exhibited a reduction in HOMA-β%, suggesting impaired basal β-cell function under fasting conditions, by 24.5% in Cd-15 ppm and 15.4% in Cd-32 ppm groups (F_2,27_ = 7.2, *p* < 0.0031). Meanwhile, Cd-exposure significantly increased the Insulinogenic Index (IGI F_2, 27_ = 32.27, *p* < 0.001), indicating a marked elevation in the insulin response relative to glucose levels in treated animals (Fig. 1B). Both the 15-ppm and 32-ppm groups had significantly higher IGI values compared to the control group (92.8% and 85.7%, *p* < 0.001).

We also assessed pancreatic β-cell functionality by measuring static glucose-stimulated insulin secretion (GSIS) in vitro (Fig. 1C). Control group islets showed progressive insulin release over 60 min, peaking at approximately 0.8 µUI/mL/islet-equivalent at 30 min. Islets isolated from the Cd-exposed groups exhibited altered phase on glucose-induced insulin secretion. The Cd-15 ppm group showed a linear response during the first 15 min (0.5 µUI/mL/islet), followed by an increased insulin release at minute 30 (1.4 µUI/mL/islet), which remains high until minute 60 (0.8 µUI/mL/islet). Meanwhile, the islets from the Cd-32 ppm group showed a higher insulin concentration from the start of glucose stimulation, reaching 2.1 µUI/mL/islet at minute 30 and remaining elevated until minute 60 (1.2 µUI/mL/islet). These results show that β-cells in Cd-exposed animals are highly sensitive to glucose-induced insulin secretion, displaying altered secretion kinetics characterized by sustained insulin release during the later stages of stimulation. The strong interaction between time and Cd dose (F_2,6_ = 55.6, *p* < 0.0001) indicates that Cd exposure significantly modifies the temporal profile of glucose-stimulated insulin secretion.

Additionally, immunofluorescence images of insulin in islets corroborated the increase in insulin expression and secretion with cadmium exposure (Fig. 1D). While insulin immunoreactivity is present in all groups, the Cd-15 ppm and, especially, the Cd-32 ppm islets exhibit a higher cytoplasmic signal.

### Proliferation and Survival of Pancreatic Islets after Cadmium Exposure

**Fig. 2:**
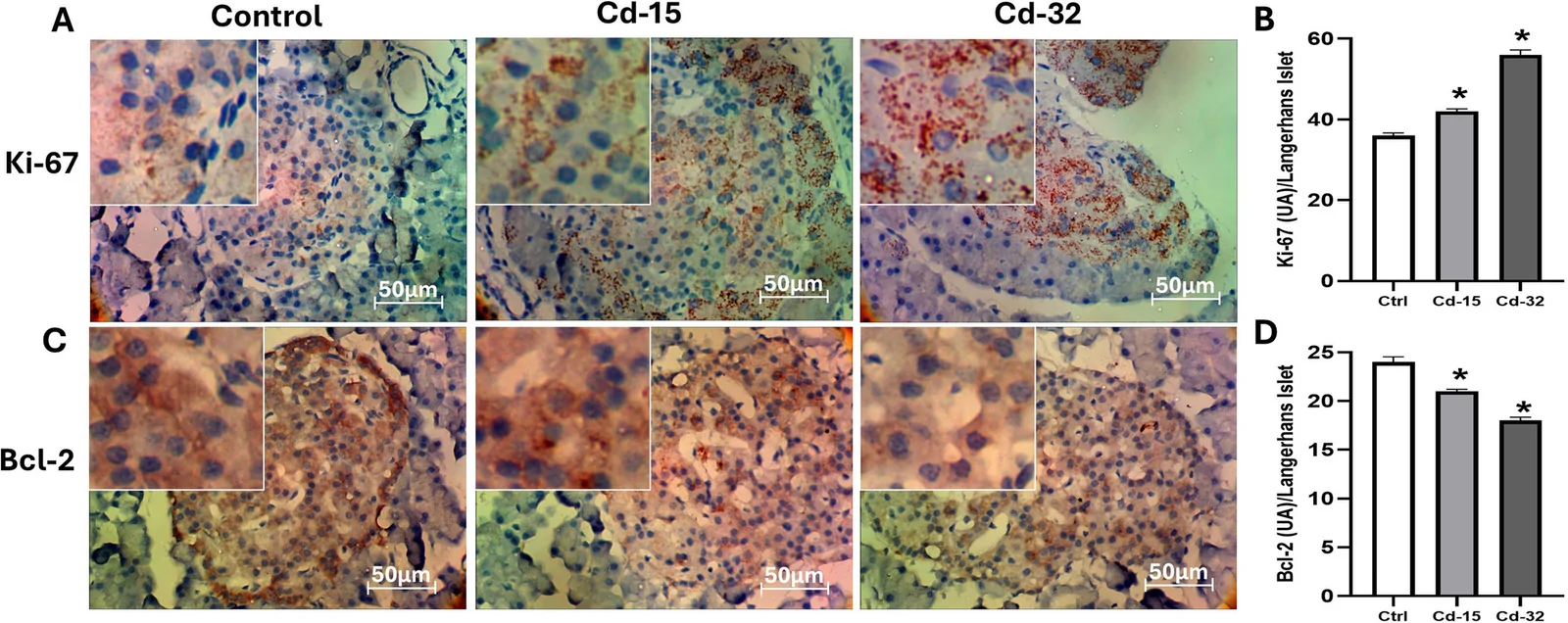
Proliferation and Survival of Pancreatic Islets after Cadmium Exposure. (**A**) Representative photomicrographs of Ki-67 expression in Langerhans islets. (**B**) Semi-quantification of Ki-67/Langerhans islet. (**C**) Representative photomicrographs of Bcl-2 expression in Langerhans islets. (**D**) Semi-quantification of Bcl-2/Langerhans islet. The data presented are the mean of the 5 repeated measurements ± SEM. Kruskal–Wallis H-test was performed, followed by a post-hoc analysis (*n* = 100, islets analyzed). (*) Indicates a significant difference at *p* ≤ 0.05

Immunohistochemistry for Ki-67 was markedly elevated in islets from rats exposed to Cd-15 ppm and Cd-32 ppm (Fig. 2A). Semi-quantification confirmed Ki-67 upregulation (Fig. 2B), increasing pixel density by 10.5% (Cd-15 ppm) and 50% (Cd-32 ppm) compared with the control group, indicating that Cd-exposure significantly induces cellular proliferation in the Langerhans islets (H = 33.56, *p* < 0.001). Conversely, the anti-apoptotic marker Bcl-2 expression decreased significantly in a dose-dependent manner in the Cd-exposure groups (Fig. 2C, D). Meanwhile, the control group displayed strong cytoplasmic Bcl-2 immunoreactivity in the Langerhans islets; the Cd-15 ppm and Cd-32 ppm groups showed diminished Bcl-2 immunoreactivity by 12.5% (*p* < 0.05) and 25% (*p* < 0.01), respectively. Results indicate that Cd exposure reduces the anti-apoptotic capacity of islet cells in a dose-dependent manner (H = 27.49, *p* < 0.001).

### Structural Changes in Insulin-Zn and Insulin-Cd Conjugates

**Fig. 3:**
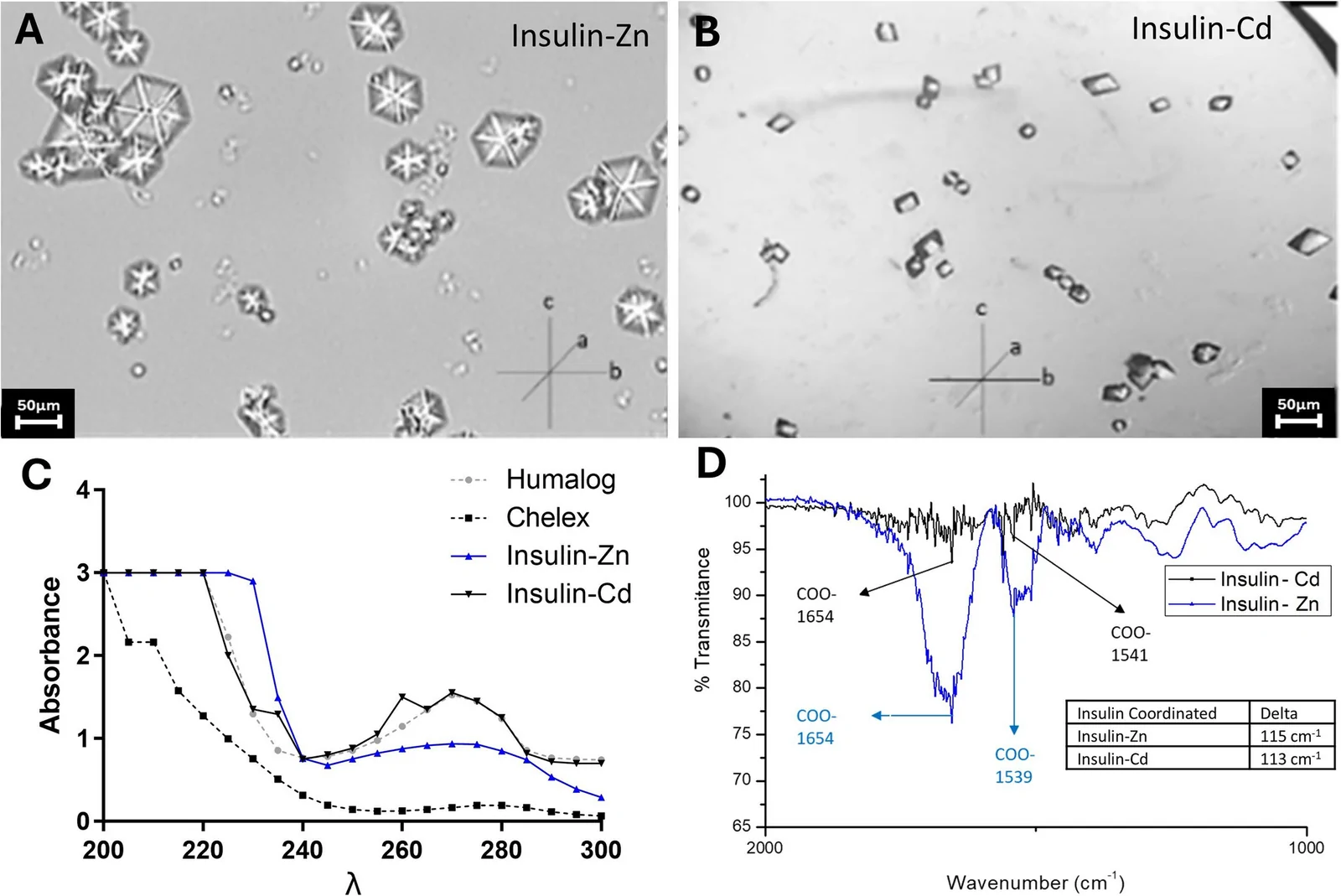
Structural changes in insulin-Zn and insulin-Cd complexes. (**A**) Representative photomicrographs of insulin-Zn crystals. (**B**) Representative photomicrographs of insulin-Cd crystals. (**C**) UV-Visible spectrum of insulin conjugates. (**D**) IR-spectrum of Insulin-Cd (black) and Insulin-Zn (blue). The photomicrograph’s magnification was 40X objective

Crystallographic morphology photomicrographs of insulin-Zn (Fig. 3A) showed regular rhomboidal crystal shapes with sharp edges and homogeneous birefringence, characteristic of native packing. Meanwhile, insulin–Cd crystals (Fig. 3B) were smaller, irregular in shape, and less defined. Although some have polyhedral geometry, most exhibit variations in symmetry and angles, suggesting less uniform packing and morphological differences that may reflect alterations in crystal organization associated with Cd incorporation into the complex.

We also characterized UV-Visible absorption spectra of insulin-Zn and insulin-Cd complexes (Fig. 3B). The UV absorption spectra of Humalog, Chelex-treated insulin, insulin–Zn, and insulin–Cd were recorded in the range of 200–300 nm to evaluate potential structural differences among formulations. All samples exhibited a strong absorption band between 200 and 225 nm, corresponding to peptide bond π→π* transitions. Humalog and insulin–Zn displayed highly similar spectral profiles, with minimal absorbance loss up to ~ 235 nm, suggesting that Zn preserves the native conformation of insulin. In contrast, Chelex-treated insulin showed a marked decrease in absorbance at 210 nm immediately after metal removal, indicating possible structural destabilization. Insulin–Cd exhibited a distinct spectral pattern, with a secondary increase in absorbance between 260 and 290 nm, consistent with conformational rearrangements affecting the microenvironment of aromatic residues such as tyrosine, phenylalanine, and tryptophan. These findings are consistent with Cd-induced structural perturbations in insulin, whereas Zn maintains a conformation more closely resembling that of the commercial preparation.

Infrared (IR) spectral analysis of insulin–Zn and insulin–Cd complexes (Fig. 3D) revealed differences in the amide absorption regions, consistent with metal-dependent conformational variations. In insulin–Zn, the amide I band appeared at 1654 cm⁻¹, while in insulin–Cd it shifted to 1641 cm⁻¹, suggesting alterations in the C = O stretching vibrations of peptide bonds and possible perturbations in secondary structure. The amide II band was located at 1539 cm⁻¹ for insulin–Zn and slightly shifted to 1541 cm⁻¹ for insulin–Cd, indicating subtle modifications in N–H bending and C–N stretching modes. Collectively, these spectral shifts and intensity variations suggest that Cd coordination induces detectable conformational rearrangements in the insulin backbone, consistent with alterations in secondary structure and potentially reducing its structural stability relative to the zinc-bound form.

To elucidate the molecular origin of these spectral differences, theoretical frequency calculations were performed for simplified metal–histidine coordination models. The Zn–His model exhibited three closely spaced imidazole ring contraction modes at 1623, 1624, and 1626 cm⁻¹ with nearly equal intensities (1.186, 1.011, 1.036), consistent with a symmetric and homogeneous coordination environment (Supplementary Fig. S1). Conversely, in the Cd–His complex, these modes shifted to 1640, 1643, and 1657 cm⁻¹ with pronounced intensity disparities (0.571, 0.783, 2.552), indicative of asymmetric binding and localized electronic perturbation of the histidine rings (Supplementary Fig. S2). The intense band at 1657 cm⁻¹ corresponds to imidazole, which interacts most strongly with Cd, supporting a less uniform coordination environment that may be associated with local structural distortion. Furthermore, while the Zn model displays a strong and sharp amide I feature at ≈ 1670 cm⁻¹—reflecting a rigid, metal-stabilized backbone—the Cd model exhibits a broader, blue-shifted amide I (~ 1672 cm⁻¹) with reduced collective coupling among backbone carbonyls, consistent with the experimentally observed spectral changes compatible with reduced conformational order in the insulin–Cd complex.

### In-Silico Analysis of Insulin-Zn and Insulin-Cd Complexes

**Fig. 4:**
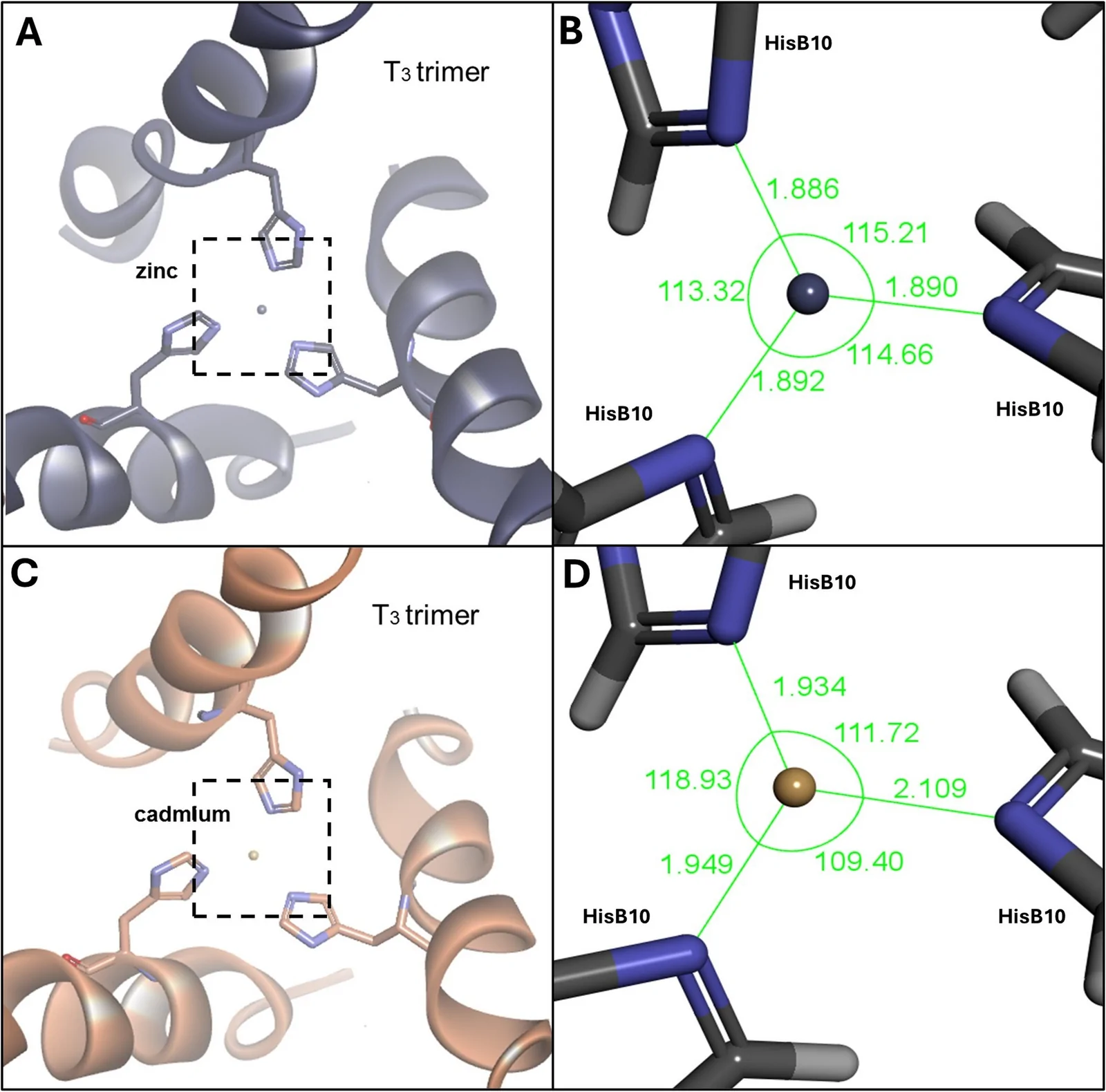
Structural coordination of Cd²⁺ or Zn²⁺ in the T₃ trimer of insulin. (**A**) The complex with Zn²⁺ shows canonical coordination mediated by the HisB10 residues of each monomer. (**B**) The bond distances (1.886–1.892 Å) and coordination angles (~ 114°) confirm an almost ideal geometry. (**C**) The Cd²⁺ ion occupies the central cavity of the trimer, adopting a similar but more expanded coordination geometry compared to the complex with Zn²⁺. (**D**) The bond distances of insulin–Cd complex (1.934–2.109 Å) and slight angular distortion (109–119°) with respect to the native zinc complex. The α-helices are represented as ribbons and the metal atoms as spheres

The coordination geometry around Cd²⁺ and Zn²⁺ ions was characterized by three nitrogen atoms (Nδ from HisB10 residues) of the insulin hexamer (Fig. 4A, C). The metal–nitrogen bond distances and angular relationships revealed distinct patterns between both metals. For Cd²⁺, the bond distances ranged from 1.93 to 2.11 Å (Fig. 4B), showing a slightly broader distribution compared with Zn²⁺, whose bonds were tightly clustered around 1.89 Å (Fig. 4A). The N–M–N angles for Cd²⁺ (109.4°, 118.9°, and 111.7°) approximate a distorted trigonal pyramidal or irregular tetrahedral geometry, indicating mild angular strain and less geometric constraint among the coordinating residues. In contrast, the angles for Zn²⁺ (114.7°, 113.3°, and 115.2°) are more uniform, producing a nearly regular tetrahedral coordination typical of zinc in metalloproteins. This variation suggests that Cd forms an asymmetric and potentially more flexible coordination environment, consistent with its larger ionic radius (0.95 Å for Cd²⁺ vs. 0.74 Å for Zn²⁺).

### In Vivo Evaluation of Insulin-Cd

**Fig. 5:**
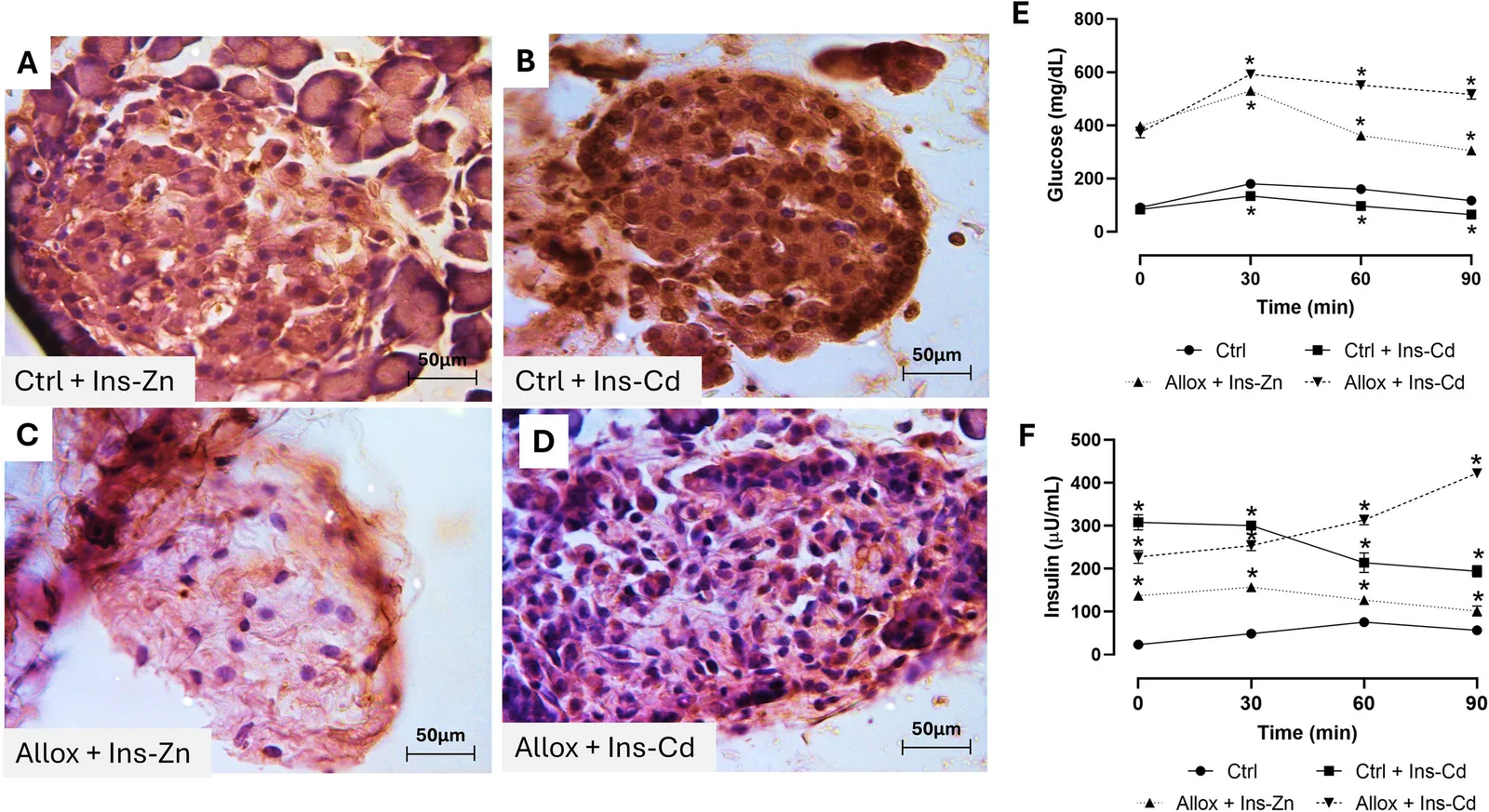
Biological Evaluation of Insulin-Cd Efficiency. (**A**) Insulin expression in Langerhans islets of the control group. (**B**) Insulin expression in Langerhans islets of the control group + Insulin-Cd. (**C**) Insulin expression in Langerhans islets of the Alloxan group + Insulin-Zn. (**D**) Insulin expression in Langerhans islets of the Alloxan group + Insulin-Cd. (**E**) Blood glucose concentration during OGTT and insulin conjugates administration. (**F**) Blood insulin concentration during OGTT and administration of insulin conjugates. The data presented are the mean of the 5 repeated measurements ± SEM. The analysis was performed by a Two-Way Repeated Measures ANOVA followed by Tukey’s post hoc test. (*) Indicates a significant difference at *p* ≤ 0.05

To determine whether insulin-Cd is biologically active, we compared its efficacy with that of insulin-Zn in control rats (non-induced) and in alloxan-induced T1D rats. First, to demonstrate that alloxan induces pancreatic damage and establishes a model of insulin dependence, we show that insulin immunoreactivity was preserved in control animals, with no major change upon administration of insulin conjugates (Fig. 5A, B). On the other hand, in the islets of animals treated with alloxan, structural damage and a reduction in insulin immunoreactivity are observed, which partially restored in diabetic rats treated with insulin-Zn (Fig. 5C). In contrast, animals treated with insulin-Cd exhibited no apparent changes were observed (Fig. 5D). The blood glucose concentrations, during OGTT shows normal behavior for the control group, 91.3 mg/dL (0 min), 190.0 mg/dL (30 min), 161.8 mg/dL (60 min), and 118.9 mg/dL (90 min). The administration of the insulin-Cd conjugate in the control group shows a small reduction in glucose compared with the control group, 85.6 mg/dL (0 min), 135.7 mg/dL (30 min), 100.6 mg/dL (60 min), and 70.4 mg/dL (90 min). Meanwhile, in the T1D model, the administration of the insulin-Zn showed that at 0 min, glucose concentration was 383.9 mg/dL, at 30 min was 564 mg/dL, at 60 min was 353 mg/dL, and at 90 min was 294 mg/dL, demonstrating the hypoglycemic effect of this conjugate. However, the T1D group treated with the insulin-Cd conjugate shows poor hypoglycemic activity, as at 0 min the glucose concentration was 390.4 mg/dL, at 30 min 600 mg/dL, at 60 min 560.3 mg/dL, and at 90 min 520.9 mg/dL, demonstrating markedly reduced hypoglycemic activity of the insulin-Cd complex under the present experimental conditions. The Two-Way repeated Measures ANOVA analysis confirms a critical failure in glucose clearance (Fig. 5E). A highly significant interaction between treatment and time was observed (F_3, 16_ = 624.12, *p* < 0.0001), driven by the persistent hyperglycemia in the Allox + insulin-Cd group compared to the successful clearance in the Allox + insulin-Zn group.

Finally, we measured serum insulin concentrations in response to an OGTT (Fig. 5F). In the intact control group, serum insulin was quantified (endogenous insulin), 22.4 ± 4.1 µUI/mL (0 min), 51.3 ± 6.6 µUI/mL (30 min), 78.4 ± 8.4 µUI/mL (60 min), and 57.2 ± 7.8 µUI/mL (90 min). In the intact control group + exogenous insulin-Cd treatment (endogenous insulin plus exogenous insulin treatment), the hormone concentration was at 0 min by 308 ± 18.3 µUI/mL, at 30 min by 301 ± 20.7 µUI/mL, at 60 min by 212 ± 15.4 µUI/mL, and at 90 min by 195 ± 14.6 µUI/mL, demonstrating the presence and minimal effect of exogen insulin. While in the Allox + insulin-Zn group, the concentration corresponded to 136 ± 12.2 µUI/mL (0 min), 154 ± 14.7 µUI/mL (30 min), 126 ± 11.8 µUI/mL (60 min), and 96 ± 10.4 µUI/mL (90 min), with a hypoglycemic effect and hormone consumption. In the Allox + insulin-Cd group, hormone quantified was 229 ± 15.5 µUI/mL (0 min), 258 ± 19.6 µUI/mL (30 min), 318 ± 22.1 µUI/mL (60 min), and 426 ± 28.5 µUI/mL (90 min), without hypoglycemic effect and insulin clearance. The statistical analysis showed a highly significant difference in total circulating insulin between the groups (F_3, 16_ = 212.85, *p* < 0.0001), supporting the conclusion that the Insulin-Cd complex exhibits markedly reduced biological activity despite its high circulating concentration.

## Discussion

In this work, we investigated the impact of chronic Cd exposure in MRL on the biochemical, structural, and functional properties of the insulin–zinc complex. Since insulin serves as the primary anabolic regulator of systemic metabolism, it orchestrates a shift from fuel mobilization to energy storage. This hormone regulates carbohydrate homeostasis by facilitating glucose uptake into tissues while simultaneously suppressing hepatic gluconeogenesis and glycogenolysis. Regarding lipid metabolism, insulin stimulates lipogenesis and the synthesis of triacylglycerols, thereby preventing the breakdown of stored fats into free fatty acids [42]. However, multiple epidemiological and experimental studies have consistently reported an association between Cd exposure and metabolic disturbances involving both carbohydrate and lipid homeostasis [15, 16, 18, 19]. Disturbances in lipid metabolism associated with Cd exposure have been reported, including hepatic steatosis, dyslipidemia, and altered lipid profiles in both human and animal models [7, 43–45]. Mechanistic studies indicate that Cd can disrupt lipid homeostasis by modulating the expression and activity of enzymes involved in lipogenesis and β-oxidation [46, 47], altering transcriptional regulation via nuclear receptors such as peroxisome proliferator-activated receptor α (PPARα) and Sterol Regulatory Element Binding Protein 1c (SREBP-1c), both of which depend on insulin signaling [7, 8, 48, 49]. Our results indicate that Cd-exposed groups (both MRL) develop dyslipidemia and, paradoxically, increase fat storage despite lipolysis, both of which are associated with insulin dysfunction. Therefore, Cd-exposed rats gain weight, increase abdominal circumference and body fat percentage, while hypertriglyceridemia with diminished HDL and elevated FFA are observed, as well as hyperglycemia and hyperinsulinemia (Table 1).

The other major metabolic function associated with insulin is glucose homeostasis, which has been studied the longest. The OGTT is the most commonly used test for this. Cadmium exposure deteriorated fasting glucose concentration in a dose-dependent manner. Post-load of glucose, Cd-exposed rats also demonstrated poor glucose management, highlighting a 30-minute time window, which yielded an F-score of 100.69, suggesting that liver glucose homeostasis was altered and that, in a minor grade, muscle glucose uptake was affected (minute 60 to 90; F-score = 26.85; Table 2). Previously, we demonstrated that environmental exposure to cadmium at the NAOEL and LOAEL doses induced hepatic insulin resistance, accompanied by metal accumulation and oxidative stress, thereby impairing glucose homeostasis [7, 8]. Buha et al. reported limited glucose uptake and utilization by skeletal muscle, thereby reducing glucose oxidation. Additionally, Cd can increase gluconeogenesis by upregulating key enzymes, while concurrently decreasing glycolytic flux, and has been shown to alter the function of glucose transporters in non-insulin-sensitive tissues, such as the intestine and kidney, potentially enhancing glucose absorption and renal glucose reabsorption. All this contributes to hyperglycemia [19].

Insulin resistance develops in a hyperinsulinemic state, as seen in rats exposed to Cd (Table 2). Significant hyperinsulinemia was observed both after fasting and 90 min post-glucose load (F = 158.98 and 155.54; *p* < 0.0001). Hyperinsulinemia during fasting and postprandial periods has been documented after chronic Cd exposure through drinking water [7, 13, 50]. This progressive hyperinsulinemia is an adaptive response by β-cells. However, prolonged β-cell overactivity can impair their ability to reduce insulin secretion over time, eventually leading to impaired function, with high hormone secretion even in euglycemic conditions [51, 52].

Our results showed a reduction in HOMA-β% in the Cd-exposed groups (Fig. 1A), suggesting impaired basal β-cell function under fasting conditions or an altered insulin secretory set point. Because Homa-β% is derived from fasting glucose and insulin concentrations, it primarily reflects basal secretory status rather than dynamic glucose-stimulated insulin release [53, 54]. In contrast, the IGI is calculated as the change in insulin relative to the change in glucose during the first 30 min of OGTT. Conversely, the increase in IGI observed in Cd-exposed animals (Fig. 1B) indicates an enhanced insulin response relative to the glucose increment during the early stage of the OGTT. Therefore, the apparent discrepancy between HOMA-β% and IGI should not be considered contradictory, as both indices evaluate different physiological aspects of β-cell performance under fasting and stimulated conditions, respectively [55]. This may reflect heightened acute secretory responsiveness via mechanisms of the canonical insulin-release pathways; however, the specific mechanisms have not been studied and must be examined in the context of Cd exposure.

At the pancreatic level, most studies report that high Cd doses, under both acute and chronic exposure, trigger β-cell death and reduce insulin production via apoptotic pathways and structural damage to the islets of Langerhans [14, 24–27]. In contrast, our in vitro study using isolated Langerhans islets (Fig. 1C) confirms that, after GSIS, Cd-exposed groups exhibit increased insulin secretion. Cadmium MRLs may induce insulin secretion through multiple, potentially overlapping mechanisms. These include modulation of membrane excitability via inhibition of K_ATP_ or voltage-gated K⁺ channels, increased Ca²⁺ influx or mobilization from intracellular stores, transient redox signaling that enhances exocytotic machinery, and possible displacement of Zn from insulin granules, facilitating their release, mechanisms primarily involved in the canonical insulin release pathway [56, 57]. Such effects could represent an early compensatory response to mild metabolic stress, preceding the β-cell dysfunction and reduced insulin output typically observed with higher or prolonged Cd exposures.

We also confirmed that the isolated islets expressed insulin by immunofluorescence (Fig. 1D). The islets from Cd-exposed rats showed strong insulin immunoreactivity and a marked increase in cell mass. Therefore, we analyzed Ki67 and Bcl-2 immunoreactivity. We observed elevated Ki67 immunoreactivity (Fig. 2A, B) and reduced Bcl-2 immunoreactivity (Fig. 2C, D) within the islets. The increase in Ki67 expression, a marker of cell proliferation [58], suggests that Cd or a metabolically altered environment may promote β-cell proliferation or increased cellular turnover, expanding the islet cell population to compensate for functional damage, thereby inducing a hyperinsulinemic response in vivo and ex vivo. Alternatively, reduced Bcl-2, a key antiapoptotic protein, may reflect increased cellular susceptibility to stress rather than purely adaptive remodeling [59]. Therefore, although the coexistence of increased Ki-67 and reduced Bcl-2 is compatible with compensatory proliferation, additional mechanistic studies are required to distinguish between adaptive remodeling and enhanced cellular vulnerability. Previously, we highlighted the role of antioxidant defense, and the HSP pancreatic defense prevents cell death associated with Cd accumulation in chronic conditions, leading to islet hyperplasia and greater secretory capacity before the onset of functional decline seen at higher or prolonged exposures [1].

Despite our clear evidence of increased insulin secretion and hyperinsulinemia in Cd-exposed groups, several factors suggest that this insulin may not be fully functional. One plausible explanation is insulin resistance, in which peripheral tissues such as the liver, skeletal muscle, and adipose tissue fail to respond adequately to circulating insulin, thereby limiting its capacity to promote glucose uptake and suppress hepatic glucose production [7, 13, 18, 19]. Additionally, a fraction of secreted insulin may exist in a structurally altered form, bound to cadmium within secretory granules, thereby impairing its receptor-binding affinity or post-receptor signaling efficacy. Thus, it is plausible that Cd–insulin complexes might alter the hormone’s bioavailability, half-life, or capacity to activate downstream pathways, ultimately contributing to hyperinsulinemia and ineffective glycemic control.

In native insulin, Zn coordinates with histidine residues (HisB10) within each monomer, stabilizing dimer and hexamer assemblies and allowing the formation of highly ordered rhombohedral crystals that facilitate storage and regulated secretion [32, 60]. This quaternary structure is insoluble and chemically stable, allowing β-cells to store insulin at high concentrations without degradation [33]. Upon exocytosis, changes in pH and extracellular zinc concentration drive the hexamer to dissociate into biologically active monomers that bind to the insulin receptor [33, 61, 62]. By contrast, Cd, although chemically similar to Zn in ionic radius, exhibits distinct coordination chemistry: it shows higher affinity for thiol-containing residues such as cysteine and methionine, while still interacting with histidines and backbone carbonyl groups. This broader binding preference, together with its more flexible coordination geometries, often results in irregular or distorted supramolecular assemblies rather than the symmetric conformations associated with Zn [34–36].

Our observations are consistent with these properties. While insulin–Zn forms typical rhombohedral crystals [63, 64], insulin–Cd forms dodecahedral or irregular structures, reflecting altered molecular-level packing interactions (Fig. 3A, B). This displacement not only changes the geometry of insulin crystals but may also stabilize alternative coordination modes, potentially through Cd–S and Cd–N bonds, which are generally stronger and longer than their Zn counterparts. Such modifications are likely to disrupt the delicate balance of insulin hexamer organization and could reduce the peptide’s structural stability and solubility. However, the present spectroscopy and computational analyses provide indirect evidence of these conformational alterations and should not be interpreted as direct atomic-resolution structural determination. The spectroscopic changes observed in both UV and FTIR analyses can be explained by the direct structural consequences of replacing Zn with Cd in the insulin hexamer. In the UV spectra (Fig. 3C), the secondary increase in absorbance between 260 and 290 nm for Insulin–Cd likely reflects alterations in the microenvironment of aromatic residues, such as tyrosine, phenylalanine, and tryptophan, which are sensitive to changes in tertiary packing and local polarity [65, 66]. In the IR spectra (Fig. 3D), the shifts in the Amide I and Amide II bands suggest modifications in hydrogen bonding patterns and backbone conformations, indicating changes in peptide bond vibrations consistent with alterations in secondary structure [66, 67]. Because Cd has a larger ionic radius and distinct coordination preferences than Zn, its incorporation may distort the histidine-coordinated core, thereby perturbing both secondary and tertiary structures. Taken together, the UV-Vus. FTIR, crystal morphology, and computational analyses provide convergent evidence supporting Cd-induced structural perturbations, although definitive characterization of these conformational changes would require high-resolution structural techniques.

The structural perturbation suggested by Cd²⁺ coordination is further supported by vibrational spectroscopy and semi-empirical computational modeling. Because the theoretical calculations were performed using simplified histidine-metal coordination models, they should be interpreted as mechanistic support for the experimental observations rather than as complete representations of the intact insulin hexamer. The experimental FT-IR band near 1540 cm⁻¹, attributed to interchain β-sheet backbone vibrations [68], exhibits a marked reduction in intensity in Cd-bound samples. This attenuation, consistent with our theoretical frequency calculations (PM7, MOPAC2016), indicates a loss of vibrational coherence and diminished β-structural content in the interchain region (S1). Normal mode analysis further reveals a decoupling of backbone vibrational modes and increased heterogeneity in histidine bands in the Cd model, consistent with a more polarized and disordered coordination field. In addition, the distinct coordination geometries of Zn²⁺ and Cd²⁺ underpin their differential effects on insulin oligomerization (Fig. 4). Our results indicate that Zn²⁺ establishes a symmetric and rigid coordination environment, thereby stabilizing the native hexameric structure. In contrast, Cd²⁺ induces heterogeneity in bond lengths and angular distortions within the coordination sphere, leading to local structural disorder. Calculated bond orders show that certain Cd–N interactions possess a greater degree of covalent character than their Zn–N counterparts, suggesting stronger but less geometrically constrained bonding, which could promote the formation of stable, non-canonical dimers under physiological pH. However, bond-order values alone are insufficient to infer thermodynamic stability, underscoring the need for complementary binding free-energy calculations and displacement assays. Collectively, these findings suggest that Cd²⁺ coordination perturbs the local topology necessary for canonical dimer formation, thereby potentially interfering with the optimal assembly and stability of the native insulin hexamer. The structural perturbation provides a plausible molecular basis for the paradoxical observation of hyperinsulinemia in Cd-exposed animals, wherein increased secretion may be accompanied by reduced functional bioactivity of the hormone.

We tested this hypothesis by administering these insulin conjugates to control and T1D model animals. Control groups showed a normal insulin expression (Fig. 5A and B). Meanwhile, alloxan induced pancreatic damage (Fig. 5C and D), as evidenced by structural damage and low insulin expression in the pancreatic islets. Our findings provide direct in vivo evidence that replacing Zn with Cd in the insulin preparation results in a marked reduction in hypoglycemic efficacy. While the insulin-Zn complex successfully improved glucose clearance during an OGTT in T1D rats, the Insulin-Cd conjugate failed to lower blood glucose levels, maintaining severe hyperglycemia (Fig. 5E). These findings support the critical role of Zn in preserving the biological architecture and function of insulin. The persistent hyperglycemia observed in the T1D + insulin-Cd group (reaching 600 mg/dL at 30 min and remaining highly elevated) indicates that the insulin-Cd complex is nonfunctional, possibly due to structural modifications. The structural perturbation likely disrupts the conformational integrity of the insulin monomer, hindering its ability to bind to and activate the insulin receptor tyrosine kinase in target tissues. Even in control rats with intact endogenous insulin, the administration of insulin-Cd produced only a minimal hypoglycemic response, further confirming its inherent biological inertness.

Perhaps the most striking finding of this study is the systemic accumulation of the Insulin-Cd complex, resulting in a profound paradox: elevated but dysfunctional insulin. In the T1D group treated with insulin-Zn, serum insulin levels peaked appropriately and then declined linearly (from 154 to 96 µUI/mL), demonstrating normal hormone consumption, receptor-mediated endocytosis, and subsequent degradation. Conversely, the insulin-Cd complex not only failed to clear glucose but progressively accumulated in the bloodstream, rising from 229 µUI/mL to a staggering 426 µUI/mL by 90 min (Fig. 5F). This accumulation indicates that the structurally altered Insulin-Cd complex may undergo altered physiological clearance. Insulin clearance is mediated by the insulin-degrading enzyme (a zinc-metalloprotease). One possible explanation is that structural modifications induced by Cd coordination alter substrate recognition by the insulin-degrading enzyme (IDE) or other clearance pathways, preventing the enzyme from recognizing and degrading the hormone and resulting in this dysfunctional hyperinsulinemic state. However, this hypothesis was not directly evaluated in the present study and therefore remains speculative.

In summary, exposure of rats to Cd MRLs led to insulin-associated dysregulation of glucose and lipid homeostasis. At the same time, there is β-cell proliferation and a loss of glucose-associated β-cell responsiveness, with overgeneration and insulin release, leading to hyperinsulinemia in vivo and ex vivo. For the first time, spectroscopic, crystal morphology, and in silico data collectively support the hypothesis that Cd can displace Zn with insulin-associated coordination sites, producing structural perturbations that may reduce its biological activity. This dual effect enhanced secretion, but potential loss of functional potency highlights a novel mechanism by which Cd could contribute to metabolic dysregulation and the early stages of insulin resistance. Our new findings provide novel insights into mechanisms that may drive metabolic dysregulation and exacerbate diabetes pathogenesis in response to environmental Cd exposure. These results emphasize the need to consider not only the quantity of insulin secreted under toxicant exposure but also its molecular integrity and bioefficiency. In conclusion, chronic Cd exposure at the experimental concentrations used in this study is associated with structural perturbations of insulin and markedly reduced biological activity, providing a plausible mechanism for Cd-induced metabolic dysfunction. 

## Limitations

The present study has some limitations. For example, direct quantification of Cd incorporation into endogenous insulin isolated from Cd-exposed animals was not performed. Likewise, structural characterization relied on complementary spectroscopic and computational approaches rather than high-resolution structural techniques. And finally, the pharmacokinetic properties and tissue distribution of the insulin–Cd conjugate were not evaluated. Nevertheless, the convergence of in vivo, in vitro, spectroscopic, and computational evidence supports the proposed mechanistic framework and provides a basis for future investigations.

## Supplementary Information

Below is the link to the electronic supplementary material.

Supplementary Material 1

## Data Availability

No datasets were generated or analysed during the current study.
