# Supplementary material for: Cadmium-Induced Structural Destabilization of the Insulin–Zinc Complex Underlies the Paradox of Elevated but Dysfunctional Insulin
Source: Biol Trace Elem Res. 2026 Jul 29;204(11):8250–66. doi: 10.1007/s12011-026-05255-7 (PMC13714130; doi:10.1007/s12011-026-05255-7)
Supplement: Supplementary file 1 [file 12011_2026_5255_MOESM1_ESM.docx]

**Supplementary Figure S1.** Infrared spectrum calculated for the insulin complex with Zn²⁺. The spectrum was obtained by semi-empirical calculations (PM7 method) in the aqueous phase using the COSMO model, and processed in Gabedit using a Gaussian convolution profile with a width at half-height of 103.3 cm⁻¹. The main bands correspond to N–H and C=O bond vibrations of the histidine residues and peptide groups involved in the coordination of the Zn²⁺ ion.

**Supplementary Figure S2.** Infrared spectrum calculated for the insulin complex with Cd²⁺. The spectrum was obtained by semi-empirical calculations (PM7 method) in the aqueous phase using the COSMO model, and processed in Gabedit using a Gaussian convolution profile with a width at half-height of 103.3 cm⁻¹. The main bands correspond to N–H and C=O bond vibrations of the histidine residues and peptide groups involved in the coordination of the Cd²⁺ ion.

**Supplementary Table S1.**

|  | Cd | Zn |
| --- | --- | --- |
| Distance M-N1 (Å) (B) | 1.949 | 1.892 |
| Distance M-N2 (Å) (F) | 2.109 | 1.890 |
| Distance M-N3 (Å) (J) | 1.934 | 1.886 |
| Bond Order M-N1 | 0.695 | 0.514 |
| Bond Order M-N2 | 0.471 | 0.516 |
| Bond Order M-N3 | 0.713 | 0.532 |
| Angle N1-Metal-N2 | 109.4 | 114.66 |
| Angle N1-Metal-N3 | 118.9 | 113.3 |
| Angle N2-Metal-N3 | 111.7 | 115.21 |

Calculated coordination parameters (bond distances, angles, and intensities) for Zn– and Cd–histidine models.
